# A phylogenetic estimate for golden moles (Mammalia, Afrotheria, Chrysochloridae)

**DOI:** 10.1186/1471-2148-10-69

**Published:** 2010-03-09

**Authors:** Robert J Asher, Sarita Maree, Gary Bronner, Nigel C Bennett, Paulette Bloomer, Paul Czechowski, Matthias Meyer, Michael Hofreiter

**Affiliations:** 1 Department of Zoology, University of Cambridge, Cambridge, UK; 2 Department of Zoology and Entomology, University of Pretoria, Pretoria, South Africa; 3 Department of Genetics, University of Pretoria, Pretoria, South Africa; 4 Department of Zoology, University of Cape Town, Cape Town, South Africa; 5 Max Planck Institute for Evolutionary Anthropology, Leipzig, Germany; 6 Department of Biology, University of York, York, UK

## Abstract

**Background:**

Golden moles (Chrysochloridae) are small, subterranean, afrotherian mammals from South Africa and neighboring regions. Of the 21 species now recognized, some (e.g., *Chrysochloris asiatica*, *Amblysomus hottentotus*) are relatively common, whereas others (e.g., species of *Chrysospalax, Cryptochloris*, *Neamblysomus*) are rare and endangered. Here, we use a combined analysis of partial sequences of the nuclear GHR gene and morphological characters to derive a phylogeny of species in the family Chrysochloridae.

**Results:**

Although not all nodes of the combined analysis have high support values, the overall pattern of relationships obtained from different methods of phylogeny reconstruction allow us to make several recommendations regarding the current taxonomy of golden moles. We elevate *Huetia *to generic status to include the species *leucorhinus *and confirm the use of the Linnean binomial *Carpitalpa arendsi*, which belongs within Amblysominae along with *Amblysomus *and *Neamblysomus*. A second group, Chrysochlorinae, includes *Chrysochloris, Cryptochloris, Huetia, Eremitalpa, Chrysospalax*, and *Calcochloris*. Bayesian methods make chrysochlorines paraphyletic by placing the root within them, coinciding with root positions favored by a majority of randomly-generated outgroup taxa. Maximum Parsimony (MP) places the root either between chrysochlorines and amblysomines (with *Chlorotalpa *as sister taxon to amblysomines), or at *Chlorotalpa*, with the former two groups reconstructed as monophyletic in all optimal MP trees.

**Conclusions:**

The inclusion of additional genetic loci for this clade is important to confirm our taxonomic results and resolve the chrysochlorid root. Nevertheless, our optimal topologies support a division of chrysochlorids into amblysomines and chrysochlorines, with *Chlorotalpa *intermediate between the two. Furthermore, evolution of the chrysochlorid malleus exhibits homoplasy. The elongate malleus has evolved just once in the *Cryptochloris-Chrysochloris *group; other changes in shape have occurred at multiple nodes, regardless of how the root is resolved.

## Background

Golden moles (Chrysochloridae) are small, burrowing mammals endemic to sub-Saharan Africa. Bronner and Jenkins [1] divide the group into nine genera and 21 species, most of which are recorded from South Africa. They have been dubbed "spectacularly autapomorphic" [2] and are among the most unusual of mammals, showing three long bones in the forearm [3], hypertrophied middle ear ossicles [4-6], and a hyoid-mandible articulation [7], among other features. They converge in many ways on the phenotype of other subterranean mammals, such as lipotyphlan moles (Talpidae), burrowing rodents (Bathyergidae), certain armadillos (*Chlamyphorus*), and marsupial moles (Notoryctidae), but lack a close phylogenetic relationship with these other groups. Rather, chrysochlorids are now understood to be part of Afrotheria, a radiation of endemic African mammals, also including hyracoids (hyraxes), proboscideans (elephants), sirenians (sea cows), macroscelidids (sengis or elephant shrews), tubulidentates (aardvarks), and tenrecids (tenrecs) [8-12].

Previous taxonomic treatments of chrysochlorids have differed not only in terms of the implied intra-familial interrelationships, but also in the number of genera used to categorize a roughly similar hypodigm of extant species. In contrast to the nine genera given by Bronner and Jenkins [1], the number of genera recognized by other authors ranges from five to seven [13-15]. Uncertainty surrounding difference among previous taxonomic treatments are likely the result of emphasis on of different types of characters that vary in phylogenetic signal.

**Figure 1:**
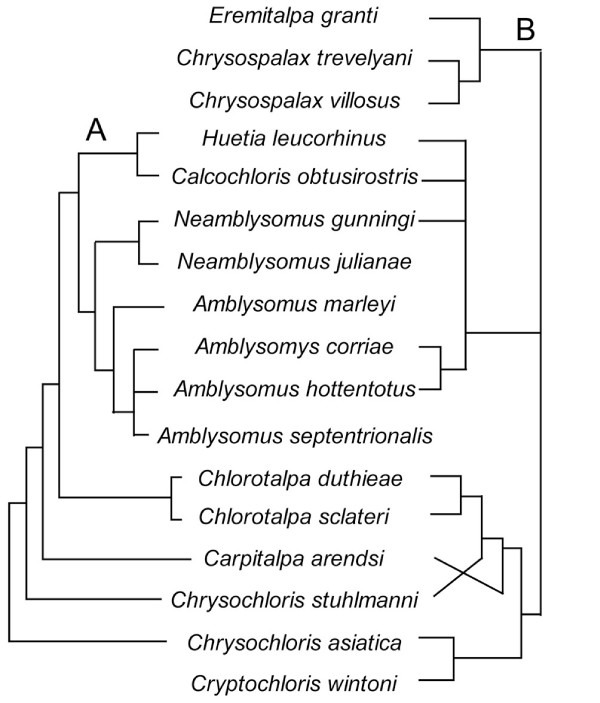
**Previously published estimates of chrysochlorid phylogeny: A) from Bronner reference seventeen: fig. nine.eleven and B) from Simonetta reference thirteen: fig. two**.

The only previous, character-based phylogenies of chrysochlorids were based on hyoid shape [7], chromosome morphology [16], and craniodental anatomy [17] from approximately a dozen species (Fig. 1A). Simonetta [13] also presented an estimate of chrysochlorid interrelationships (Fig. 1B), but did not base his  on an explicit matrix of morphological or molecular data. These published trees contradict at least some genus-level relationships frequently implied in the literature [18]. For example, both Simonetta [13] and Bronner [17] imply paraphyly of the genus *Chrysochloris*, consisting of the Cape golden mole, *Chrysochloris asiatica*, and a tropical congener, *C. stuhlmanni *(Fig. 1). The geographical disparity of *Chrysochloris *populations---with over 3000 km separating the Western Cape province of South Africa (*C. asiatica*) from the region surrounding Lake Victoria (*C. stuhlmanni*)---lends itself to the possibility of genus-level paraphyly. Similarly, occurrences within what previous authors [1] referred to as *Calcochloris *are separated by at least 2000 km: *C. obtusirostris *in southern Mozambique and *"C." leucorhinus *in the Democratic Republic of the Congo and Angola. Other classifications of *leucorhinus *(e.g., in the genus *Chlorotalpa *[15] along with *C. duthieae *and *C. sclateri *from South Africa) do not improve the geographic cohesiveness of this taxon. Hence, the monophyly of some chrysochlorid genera has not been consistently supported [13,17] and deserves to be tested.

Here, we present a phylogenetic analysis of the Chrysochloridae based on 145 morphological characters from the cranium, dentition, and skeleton, combined with approximately 700-900 bases from exon 10 of the nuclear Growth Hormone Receptor (GHR) gene for 18 of the group's currently recognized 21 species. This locus has frequently been applied to questions regarding afrotherian systematics [e.g., [19,20]], and along with other nuclear sequences supports a sister-group relationship between chrysochlorids and tenrecids [e.g., [10]]. This study attempts to provide an evolutionary basis for chrysochlorid classification and thereby aid the process of understanding chrysochlorid systematics and evolution.

## Results

**Figure 2:**
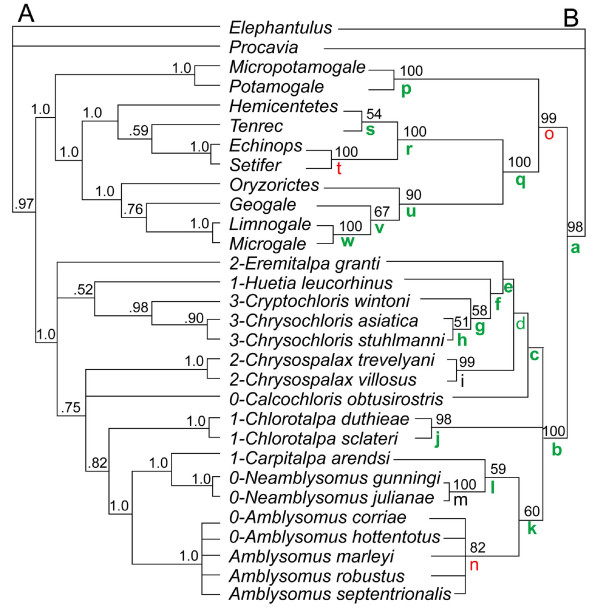
**Optimal phylogenetic trees (branch lengths arbitrary) derived from combined GHR-morphology-indel dataset using A) MrBayes with HKY+G model for GHR partition (majority rule consensus of 9901 post-burnin trees from run #1; support indices represent Bayesian posterior probabilities) and B) MP with equally weighted data (strict consensus of 8 trees at 957 steps; support indices represent bootstrap values from 500 pseudoreplicates of a simple addition sequence, not reported under 50)**. Numbers adjacent to chrysochlorid taxa represent coding of mallear head, corresponding to the character states illustrated in Fig. 5 (data missing for some species of *Amblysomus*). Colored letters adjacent to clades correspond to the partitioned branch support analysis presented in Table 1. Green = positive HBS, red = negative HBS, black = additive HBS (see Table 1).

The Chrysochloridae and several clades within it are recovered with high support from our Bayesian and MP analyses of the combined morphology-GHR dataset (Fig. 2), including the genera *Amblysomus, Neamblysomus, Chlorotalpa*, and *Chrysospalax*. The genus *Chrysochloris*, including both *C. stuhlmanni *from near Lake Victoria and *C. asiatica *from the Western Cape, is also supported as monophyletic with high Bayesian but low MP support (Fig. 2). Both MP and Bayesian analyses support the sister taxon status of *Cryptochloris *and *Chrysochloris*, again with Bayesian support higher than that from MP. In agreement with both Simonetta (reference thirteen: fig. 2) and Bronner [17], "*Calcochloris" leucorhinus *does not form a clade with *Calcochloris obtusirostris *in either the Bayesian or MP analyses (Fig. 2), making *Calcochloris *(sensu [17]) paraphyletic. For this reason, we regard *Huetia *(previously used as a subgenus [1]) as the appropriate generic name for the species *leucorhinus*.

The association of *Carpitalpa arendsi *with *Neamblysomus *(Fig. 2) is consistent with the recognition of *Carpitalpa *as a distinct genus [1], in contrast to previous classifications which assigned *arendsi *to either *Chlorotalpa *[18] or *Amblysomus *[14]. *Amblysomus *and *Chlorotalpa *comprise successively distant sister taxa to the *Carpitalpa-Neamblysomus *clade following our optimal Bayesian trees; MP agrees concerning *Amblysomus *but does not consistently resolve the position of *Chlorotalpa*.

**Figure 3:**
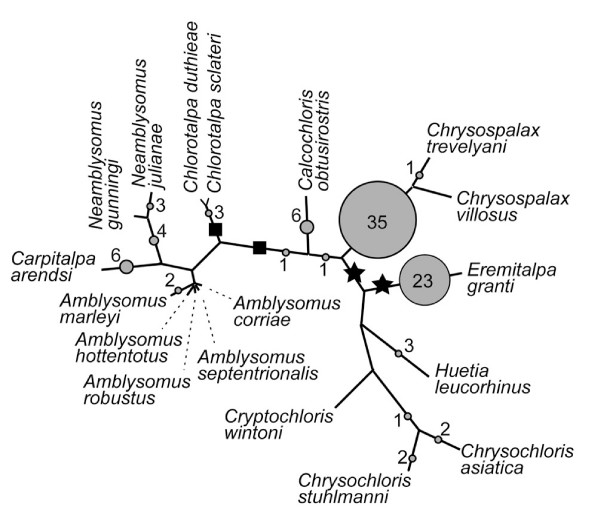
**Unrooted consensus network consistent with optimal Bayesian and MP topologies shown in Fig. 2**. Branch lengths are proportional to number of changes obtained in MP tree #2 [see Additional file 1] derived from the combined dataset, using assigned branch length in PAUP and assuming accelerated transformations (ACCTRAN). Dashed lines for *Amblysomus *are diagrammatic only. Squares indicate the two root positions (i.e., where Tenrecidae attaches) favored by 4 of 8 optimal MP trees (Fig. 2B), stars those favored by the majority of Bayesian post-burnin topologies (Fig. 2A). The branch leading to Tenrecidae is 106 steps; the longest intra-chrysochlorid branches are those leading to *Chrysospalax *and *Eremitalpa *at 15 steps each. Circles denote nodes to which the 100 randomly generated outgroup taxa were attracted, and are proportionately larger when more random outgroups attach at that node (indicated by the number within/adjacent to each circle). Seven of the randomly generated outgroups did not yield a resolved position for the root.

Bayesian analyses do not resolve the chrysochlorid root, but favor *Eremitalpa *and a *Huetia-Chrysochloris-Cryptochloris *group as the basal-most taxa (Fig. 2A). Results from MP also show an unresolved root, but differ by reconstructing chrysochlorines as monophyletic (Fig. 2B). In half of the optimal trees, MP places the root between monophyletic chrysochlorines and amblysomines plus *Chlorotalpa*, or with *Chlorotalpa *itself as the basal-most clade, also with a monophyletic Chrysochlorinae and Amblysominae. Despite this difference, the unrooted topologies supported by Bayesian and MP algorithms are mutually consistent (Fig. 3).

To further examine the position of the root, and to infer the node potentially most susceptible to long branch attraction [21], we tested the affinities of 100 randomly-generated taxa, each of which was used in turn as the outgroup for an MP analysis of our 18 chrysochlorid taxa. As depicted in Fig. 3, 36 of these outgroups rooted the chrysochlorid tree at or within Chrysospalax, 23 at Eremitalpa, 7 at or within Neamblysomus, 6 at Carpitalpa, 6 at Calcochloris, and 5 at or within Chrysochloris. Seven random outgroups yielded topologies that lacked a resolved root in a majority rule consensus of the optimal trees for that analysis. The remaining root-positions exhibited by the randomly-generated outgroups occurred at frequencies between 1-4 (Fig. 3). Hence, the equally-weighted MP analysis of GHR, morphology, and indels (Fig. 2B) does not root the chrysochlorid tree near the branches to which the greatest number of randomly generated outgroups are attracted, but the Bayesian analysis does. As previously noted, the optimal MP trees show a root at either Chlorotalpa or between chrysochlorines and amblysomines+Chlorotalpa. Only four of the 100 randomly-generated outgroups rooted the chrysochlorid tree adjacent to these nodes (Fig. 3). Bayesian analyses, in contrast, more frequently rooted the chrysochlorid topology at or near one of the longest branches within the chrysochlorid tree, albeit without strong support values.

Overall, both MP and Bayesian algorithms agree on the basic branching pattern within chrysochlorids (Fig. 3): an *Amblysomus-Neamblysomus-Carpitalpa *group, a *Chrysospalax-Calcochloris-Chrysochloris-Cryptochloris-Huetia-Eremitalpa *group, with the two species of *Chlorotalpa *between the two. However, we acknowledge that support indices for most basal nodes remain low, and we cannot yet be certain about the position of the chrysochlorid root.

**Figure 4:**
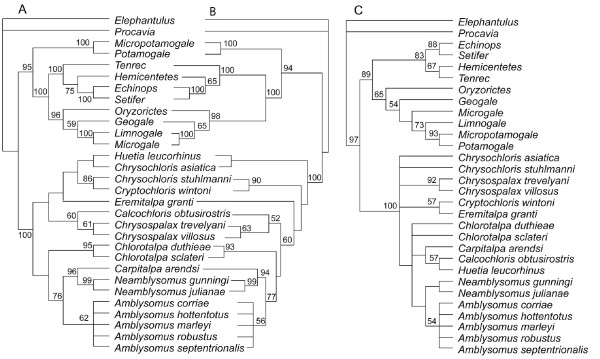
**Optimal trees derived from partitioned analyses of A) GHR-indels using MP (1 tree at 600 steps), B) GHR using the likelihood model HKY85+G (score of optimal tree = ln -4442.86452), and C) morphology using MP (strict consensus of 16 trees at 331 steps)**. MP support values (A and C) are based on 500 bootstrap pseudoreplicates of a simple addition sequence; likelihood support values (B) are based on 291 bootstrap pseudoreplicates of an as-is addition sequence, also using the HKY85+G model.

Fig. 4 shows the trees supported by the GHR (Figs. 4A, B) and morphology (Fig. 4C) partitions examined separately. Aspects of non-chrysochlorid afrotherian phylogeny are discussed elsewhere [20]. For chrysochlorids, the molecular and morphological partitions agree on supporting *Amblysomus, Chrysospalax, Neamblysomus*, and Chrysochloridae. Morphology alone does not resolve most supra-generic clades, and where resolution exists, support is weak. It does resolve a *Neamblysomus-Amblysomus *clade, but without *Carpitalpa arendsi *as portrayed in the combined (Fig. 2) and GHR (Figs. 4A, B) datasets. Morphological data weakly support an *Eremitalpa-Cryptochloris *clade and a *Chlorotalpa-Carpitalpa-Calcochloris-Huetia-Amblysomus-Neamblysomus *clade, groupings which collapse when MP is relaxed by one step. Nevertheless, as documented elsewhere [22-24], the performance of a given partition in isolation should not prevent its combination with other datasets. As shown in the distribution of Hidden Branch Support values [HBS, see Table 1 and [22-24]], for the 19 clades that are present in both the combined (Fig. 2B) and GHR-only (Fig. 4A) MP trees, 12 show increased branch support with addition of morphology and indels, 4 do not change, and 3 show decreased branch support.

**Table 1:** MP branch supports for clades (lettered as in Fig. 2B) across combined ("comb"), GHR, morphology ("morph"), and indel partitions.

node from fig 2B	comb	comb WO	comb BS	GHR	GHR WO	GHR BS	morph	morph WO	morph BS	indel	indel WO	indel BS	HBS
a	957	970	13	591	593	2	331	340	9	8	8	0	2

b	957	1004	47	591	605	14	331	344	13	8	8	0	20

c	957	958	1	591	592	1	333	331	-2	8	8	0	2

d	957	958	1	593	591	-2	334	331	-3	8	8	0	6

e	957	959	2	591	592	1	333	331	-2	8	8	0	3

f	957	958	1	591	592	1	336	331	-5	8	8	0	5

g	957	958	1	593	591	-2	333	331	-2	8	8	0	5

h	957	958	1	593	591	-2	331	331	0	8	8	0	3

*i*	*957*	*962*	*5*	*591*	*592*	*1*	*331*	*334*	*3*	*8*	*9*	*1*	*0*

j	957	962	5	591	594	3	331	331	0	8	8	0	2

k	957	958	1	591	593	2	334	332	-2	8	8	0	1

l	957	958	1	591	595	4	335	331	-4	8	8	0	1

*m*	*957*	*963*	*6*	*591*	*596*	*5*	*331*	*332*	*1*	*8*	*8*	*0*	*0*

**n**	**957**	**958**	**1**	**591**	**592**	**1**	**331**	**332**	**1**	**8**	**8**	**0**	**-1**

**o**	**957**	**966**	**9**	**591**	**596**	**5**	**331**	**337**	**6**	**8**	**8**	**0**	**-2**

p	957	990	33	591	615	24	331	338	7	8	8	0	2

q	957	978	21	591	613	22	338	331	-7	8	8	0	6

r	957	971	14	591	600	9	331	335	4	8	8	0	1

*s*	*957*	*958*	*1*	*593*	*591*	*-2*	*331*	*334*	*3*	*8*	*8*	*0*	*0*

**t**	**957**	**970**	**13**	**591**	**601**	**10**	**331**	**335**	**4**	**8**	**8**	**0**	**-1**

u	957	963	6	591	597	6	336	331	-5	8	8	0	5

v	957	959	2	591	592	1	337	331	-6	8	8	0	7

w	957	968	11	591	601	10	335	331	-4	8	8	0	5

Numbers below each partition name represent optimal treelength for clade in left-most column; column "WO" indicates optimal treelength without that clade; column "BS" indicates branch support for that clade from partition (i.e., partition WO - partition). Hidden Branch Support (HBS, see [22-24]) represents the difference between combined branch support and the sum of branch support from each partition. Positive HBS (plain text) indicates more support from combined analysis than from sum of partitions; negative HBS (bold) indicates support for clade lower in combined analysis relative to sum of partitions; zero HBS (italic) indicates additive branch support (support for clade the same in combined and in sum of partitions).

## Discussion

As noted above, the morphological partition alone does not recover most suprageneric clades of the combined-data analysis, and weakly disagrees with many aspects of the GHR tree (Fig. 4). However, it has been demonstrated that a given clade may receive support from a combined dataset even when that clade lacks support from every partition analyzed in isolation [22,23]. For example, at high taxonomic levels within placental mammals, morphology (among other partitions) has been shown to contribute positively to clade support in a combined analysis, whether or not that clade is present in a tree derived from morphological data alone [24]. For the present dataset, the addition of the morphological partition improves branch support for the majority of nodes present in both the optimal MP and Bayesian topologies of the combined dataset (Fig. 2; Table 1). This observation is consistent with the observation made elsewhere [22-24], based on independent datasets, in which interaction between partitions can increase support beyond the sum of branch supports obtained from partitions in isolation (i.e., "hidden support" of [22]). Hence, we base our discussion on the combined-data tree.

Our results are partly congruent with the suggestion of Bronner and Jenkins [1] that chrysochlorids may be divided into two clades: Amblysominae and Chrysochlorinae, although their content is different according to our results. We place *Amblysomus, Neamblysomus*, and *Carpitalpa arendsi *in Amblysominae. The Chrysochlorinae of Bronner and Jenkins [1], excluding *Carpitalpa *and *Chlorotalpa*, is a potentially monophyletic clade (Figs. 2B, 3), and is weakly supported as such by our MP analyses of the combined data (Fig. 2B). However, additional data are necessary to address the possibility that the chrysochlorid root falls within this group, possibly near *Eremitalpa *(Fig. 2A). For ease of discussion, we use the terms amblysomines and chrysochlorines as defined above, but acknowledge that the latter term may be rendered paraphyletic with further phylogenetic scrutiny.

### Morphological character evolution

**Figure 5:**
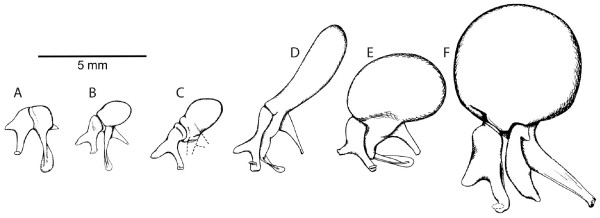
**Malleus and incus morphology among golden moles**. A) *Amblysomus hottentotus *redrawn from Mason reference six: fig. one d; B) *Chlorotalpa sclateri *redrawn from Mason reference five: fig. three b; C) *Carpitalpa arendsi *redrawn from Mason reference five: fig. three c; D) *Chrysochloris asiatica *redrawn from Mason reference six: fig. 1e; E) *Eremitalpa granti namibensis *redrawn from Mason reference six: fig. one f; F) *Chrysospalax villosus *redrawn from Mason reference four: fig. seven b. For the purposes of this study, malleus shape is coded using four states: 0-small (head of malleus does not exceed manubrium mallei in longest dimension, as in A); 1-enlarged (head of malleus similar to manubrium mallei in longest dimension, as in B and C); 2-enlarged and globular (head of malleus is pea-shaped and exceeds length of manubrium mallei, as in E and F); 3-club shaped (head of malleus is elongate and over twice the length of the manubrium mallei, as in D). Scale bar = 5 mm.

**Figure 6:**
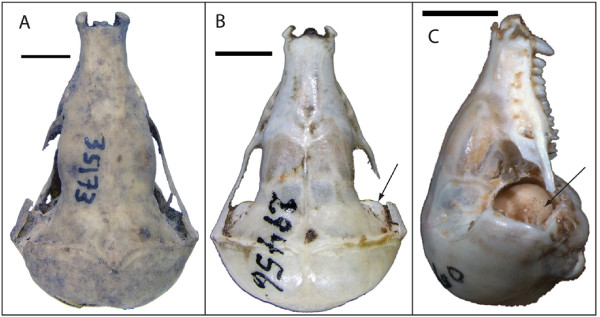
**Dorsal (A, B) and lateral (C) views of the skull in *Calcochloris obtusirostris *(A, ZMB 35173), *Chrysochloris stuhlmanni *(B, ZMB 29456), and *Eremitalpa granti *(C, TM 14060)**. Arrow in B points to temporal bulla; arrow in C points to exposed mallear head. Note lack of temporal bulla in A and enlarged epitympanic recess, housing exposed malleus, in C. Scale bars = 5 mm.

As noted previously, chrysochlorids share a number of features (e.g., three forearm long-bones, hyoid-mandible articulation, hypertrophied malleus) seldom seen elsewhere among living mammals. In the case of ossicular morphology, considerable variation exists across chrysochlorid species [4-6,25,26]. *Amblysomus, Neamblysomus*, and *Calcochloris *show a relatively small malleus (Fig. 5A), unlike the elongate, club-shaped ossicle seen in *Chrysochloris *and *Cryptochloris *(Fig. 5D). The mallear head is enlarged and globular in *Eremitalpa *(Fig. 5E) and *Chrysospalax *(Fig. 5F), and is also globular but only slightly enlarged (relative to *Amblysomus*) in *Chlorotalpa *(Fig. 5B), *Huetia*, and *Carpitalpa *(Fig. 5C). Those taxa with a club-shaped malleus all possess a substantial bulge in the posterior aspect of their orbitotemporal fossa, known as the temporal bulla, which serves as a dorsal continuation of the epitympanic recess for housing the enlarged malleus (Fig. 6). The globular malleus of *Eremitalpa *is sufficiently large that it too results in an externally visible temporal bulla (Fig. 6C). In the other taxa with a globular malleus (*Chlorotalpa, Huetia*, and *Carpitalpa*), the ossicle is smaller and does not result in an externally distinct temporal bulla.

There are a number of other variations on chrysochlorid mallear morphology not accounted for in the current morphological matrix, such as orientation of the manubrium mallei, shared for example in *Eremitalpa *and *Chrysochloris *but not in *Chrysospalax *(Fig. 5; M. Mason pers. commun. and [4-6]). Here, we focus on size and shape of the mallear head, which has figured prominently in previous classifications. This region guided the classification of Simonetta [13], who stated " [mallear] morphology pointed to three divergent evolutionary trends, so that the family could be ... divided into three subfamilies" (p. 33 of ref. [13]). He further implied the intuitive view that the "normal" (i.e., small) malleus of *Amblysomus *is primitive, and inferred a "morphological sequence" from *Amblysomus *to the taxa with a globular malleus to the "extreme" club-shape observed in *Chrysochloris*.

Interestingly, the taxa most frequently inferred as basal by Bayesian analyses possess an enlarged, globular malleus (i.e., *Eremitalpa*), a slightly enlarged malleus (*Huetia*), or an elongate malleus (*Chrysochloris *and *Cryptochloris*). If such a taxon occupies the base of the chrysochlorid tree, then Simonetta's intuition about the character evolution of the chysochlorid malleus is wrong: taxa with an unenlarged malleus (*Amblysomus, Neamblysomus, Calcochloris*) occupy relatively nested branches. In contrast, the root supported by four of the eight MP analyses (Fig. 3) shows *Calcochloris obtusirostris *(with a small malleus) at the base of a monophyletic Chrysochlorinae, which forms the sister taxon of a *Chlorotalpa-*amblysomine clade. This scenario is potentially consistent with the view that a small malleus (Fig. 5A) characterized the basal-most living chrysochlorids, with enlargement occurring independently within each subfamily. Most phylogenies of Afrotheria support a tenrec-golden mole association [10,11], and one would expect that the chrysochlorid common ancestor with other afrotherians would have had relatively small ear ossicles. Their scant fossil record [27,28] indicates that Miocene chrysochlorids lacked a temporal bulla and therefore would not have had a mallear head of the kind seen in *Chrysochloris *(Fig. 5D), *Eremitalpa *(Fig. 5E), or *Chrysospalax *(Fig. 5F).

Previous discussions of mallear evolution in chrysochlorids have also noted the high probability of homoplasy in this region [4,5,26]. While we cannot yet resolve the position of the chrysochlorid root, all of our optimal trees agree with recent authors that mallear enlargement has not occurred in a simple progression from small, to globular, to elongate. The paraphyly of chrysochlorids with an enlarged, globular malleus indicates the presence of homoplasy in the occurrence and direction of ossicular enlargement.

**Figure 7:**
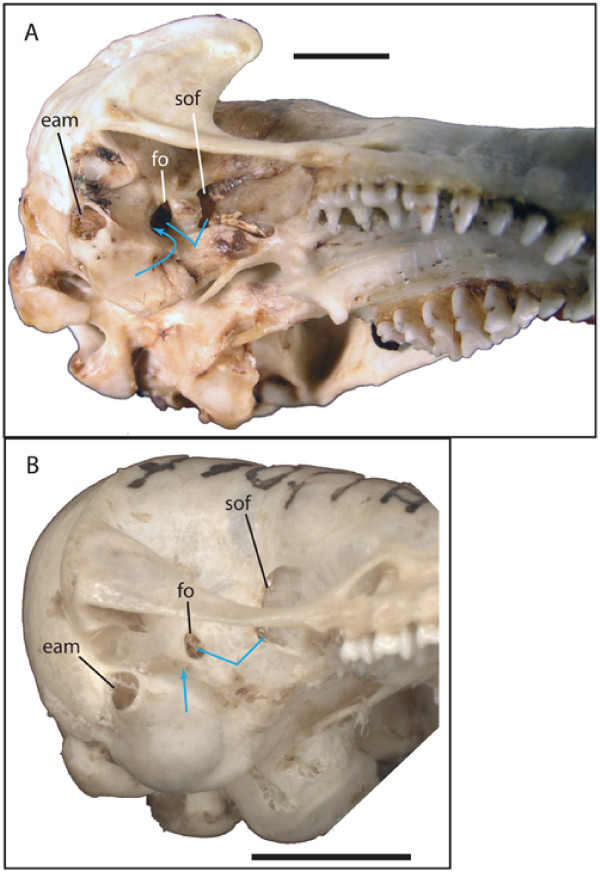
**Ventrolateral views of skulls of *Chrysospalax trevelyani *(A, TM 40501) and *Calcochloris obtusirostris *(B, ZMB 85341, image reversed)**. Curved blue arrow in A represents foramen for stapedial ramus inferior subsumed within foramen ovale; straight blue arrow in B represents foramen for stapedial ramus inferior distinct from foramen ovale. Note also distance between sphenorbital fissure and foramen ovale in B exceeding maximum length of foramen ovale aperture, whereas in A the distance between the two is similar to maximum length of foramen ovale. Abbreviations are "eam" = external auditory meatus, "fo" = foramen ovale, "sof" = sphenorbital fissure. Scale bars = 5 mm.

Based on the phylogeny in Fig. 2, a few additional morphological characters optimize with relatively low homoplasy across chrysochlorids. The position of the foramen ovale relative to the foramen for the inferior ramus of the stapedial artery (Fig. 7) shows relatively little homoplasy in the optimal MP and Bayesian trees: the two are confluent in chrysochlorines (except *Calcochloris*) and distinct in amblysomines and *Chlorotalpa*. The position of foramen ovale relative to the sphenorbital fissure (Fig. 7) also distinguishes most members of the two groups: in amblysomines and *Chlorotalpa sclateri *they are separated in the ventral part of the temporal fossa, whereas in *Chlorotalpa duthieae *and most chrysochlorines (but not *Huetia *or *Calcochloris*) they are situated close together.

**Figure 8:**
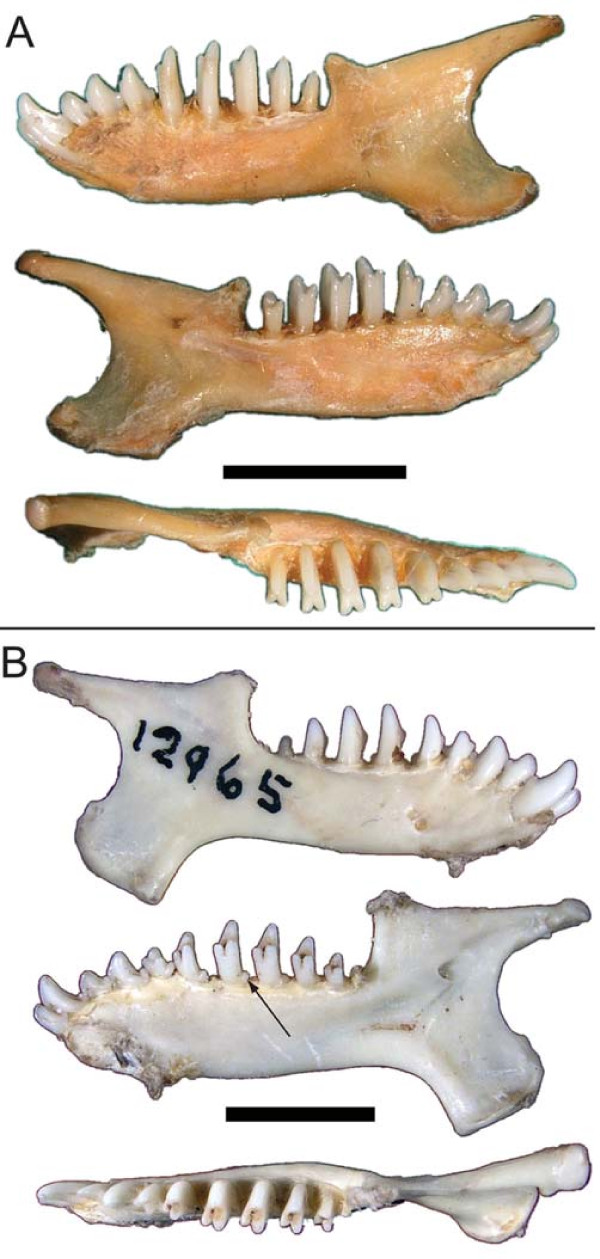
**Mandibles of *Huetia leucorhinus *(A, ZMB 31505) and *Carpitalpa arendsi *(B, TM 12965)**. Arrows in B indicate talonid on p4 in *Carpitalpa*, present throughout the lower toothrow and absent in premolars and molars of *Huetia*. Scale bars indicate 5 mm.

Dentally, most chrysochlorines (except for *Chrysospalax*) lack talonids on their lower ultimate premolar (Fig. 8), whereas these are present in amblysomines (except for *Neamblysomus julianae*) and *Chlorotalpa*. Talonids on the molars are similarly lacking in chrysochlorines (except *Chrysospalax *and *Chrysochloris stuhlmanni*), but present in amblysomines (except *Neamblysomus*) and *Chlorotalpa*. The overall number of teeth in each jaw quadrant---10 in those taxa with a full complement of three molars and 9 in taxa with just two---has also figured prominently in previous chrysochlorid classifications. However, reduction of molars is restricted to *Amblysomus, Calcochloris*, and is variable in *Neamblysomus*. As such, this feature is not diagnostic for the supra-generic clades indicated in this study.

## Conclusions

Our data support the validity of *Carpitalpa arendsi*, the removal of the equatorial species *leucorhinus *from *Calcochloris, Chlorotalpa*, and *Amblysomus*, the integrity of most chrysochlorid genera, such as *Chrysochloris *including species from both equatorial Africa (*C. stuhlmanni*) and the Western Cape province of South Africa (*C. asiatica*), and the association of *Cryptochloris *with *Chrysochloris *[40] and of *Amblysomus *with the *Neamblysomus-Carpitalpa *clade. We amend previous chrysochlorid taxonomies by using *Huetia *as the appropriate genus-level designation for *H. leucorhinus*. Both MP and Bayesian analyses support a similar unrooted topology, with amblysomines as defined above and chrysochlorines consisting of *Chrysochloris, Cryptochloris, Huetia, Chrysospalax*, and *Calcochloris*. *Chlorotalpa *is favored as the sister taxon to amblysomines in Bayesian analyses; whereas in the consensus of optimal MP trees, *Chlorotalpa *appears in an unresolved trichotomy with monophyletic amblysomines and chrysochlorines (Fig. 2B). Bayesian analyses weakly support a root within Chrysochlorinae, near *Eremitalpa *or a *Chrysochloris-Cryptochloris-Huetia *clade. As noted above, MP yields a trichotomy at the root. The fact that an intra-chrysochlorine placement of the root in some of our analyses corresponds with the region of the tree that frequently attracts randomly generated outgroup taxa leads us to regard the position of the chrysochlorid root as still unresolved and in need of further study.

An incremental, small-to-large evolution of one of the most peculiar chrysochlorid features---the enlarged malleus---is not supported by our analysis. According to topologies supported by Bayesian techniques, chrysochlorid species with a small malleus (*Amblysomus, Neamblysomus, Calcochloris*) are nested, not basal, within the chrysochlorid radiation. However, optimal MP topologies cannot rule out a small malleus characterizing the basal-most chrysochlorid branch. Furthermore, and regardless of the position of the root, change in mallear size has occurred multiple times during the history of this insectivoran-grade radiation of afrotherian mammals.

## Methods

We obtained new GHR sequences of 17 chrysochlorid species and appended them to GHR alignment #1 of Asher and Hofreiter [[20]; see Additional file 1]. As detailed in Table 2, skulls from the collections of the Museum für Naturkunde Berlin (ZMB) and the Transvaal Museum Pretoria (TM) comprised source material for five of these taxa. For these specimens we followed the DNA extraction procedure described in [20]. To control for potential sequencing artifacts, we followed [20] and [29]. Additional file 2 shows individual BLAST results for GHR sequence fragments derived from museum skulls. In every case, the closest match on GenBank was to the existing chrysochlorid (*Chrysospalax trevelyani *AF392877 from [30]), with a percent similarity ranging from 91-100. In most cases the match was not identical, either to the GenBank sequence or across our samples. Given the fact that some regions of GHR exon 10 are highly conserved across mammals and the relatively short length of our amplified fragments (ca. 60-160 bp), it is not surprising that a small number match entirely. Overall, we view this result as evidence that our sequences are genuine and not influenced by contamination.

**Table 2:** List of Genbank accession numbers and source material for DNA analysis.

clade	genus	species	source	accession
Macroscelididae	*Elephantulus*	*rufescens*	Malia et al. 2002	AF392876

Hyracoidea	*Procavia*	*capensis*	Malia et al. 2002	AF392896

Tenrecidae	*Echinops*	*telfairi*	Malia et al. 2002	AF392889

	*Geogale*	*aurita*	Asher & Hofreiter 2006	DQ202287

	*Hemicentetes*	*semispinosus*	Asher & Hofreiter 2006	DQ202288

	*Microgale*	*talazaci*	Malia et al. 2002	AF392885

	*Microgale ("Limnogale")*	*mergulus*	Asher & Hofreiter 2006	DQ202289

	*Micropotamogale*	*lamottei*	Asher & Hofreiter 2006	DQ202290

	*Oryzorictes*	*talpoides*	Malia et al. 2002	AF392886

	*Potamogale*	*velox*	Asher & Hofreiter 2006	DQ202291

	*Setifer*	*setosus*	Asher & Hofreiter 2006	DQ202292

	*Tenrec*	*ecaudatus*	Malia et al. 2002	AF392890

Chrysochloridae	*Amblysomus*	*corriae*	**TM 39451 tissue**	GU904406

	*Amblysomus*	*hottentotus*	**ZMB 3919 skull**	GU904407

	*Amblysomus*	*marleyi*	**tissue**	GU904408

	*Amblysomus*	*robustus*	**TM 41661 issue**	GU904409

	*Amblysomus*	*septentrionalis*	**TM 42135 tissue**	GU904410

	*Huetia ("Calcochloris")*	*leucorhinus*	**tissue**	GU904412

	*Calcochloris*	*obtusirostris*	**ZMB 12945 skull (syntype)**	GU904411

	*Carpitalpa*	*arendsi*	**tissue**	GU904413

	*Chlorotalpa*	*duthieae*	**TM 39456 tissue**	GU904414

	*Chlorotalpa*	*sclateri*	**TM 39439 tissue**	GU904415

	*Chrysochloris*	*asiatica*	**TM 41985 tissue**	GU904416

	*Chrysochloris*	*stuhlmanni*	**ZMB 29456 skull (syntype)**	GU904417

	*Chrysospalax*	*trevelyani*	Malia et al. 2002	AF392877

	*Chrysospalax*	*villosus*	**DM 7474 tissue**	GU904418

	*Cryptochloris*	*wintoni*	**TM 8235 skull**	GU904419

	*Eremitalpa*	*granti granti*	**TM 8248 skull**	GU904420

	*Neamblysomus*	*gunningi*	**TM 40766 tissue**	GU904421

	*Neamblysomus*	*julianae*	**TM 40126 tissue**	GU904422

Accession numbers refer to Genbank codes. DM, TM, and ZMB refer to the collections of the Durban Natural Science Museum, Transvaal Museum Pretoria, and the Zoologisches Museum Berlin (= Museum für Naturkunde), respectively. Novel GHR sequences are identified in bold.

The remainder of our chrysochlorid sample derived from work undertaken at the Universities of Pretoria and Cape Town (Table 2). Methods for obtaining partial GHR sequences from these taxa are as follows: genomic DNA was extracted from frozen or ethanol-preserved tissues using standard phenol-chloroform procedures [31]. A 767 bp fragment was amplified for 16 samples using the polymerase chain reaction (PCR, [32]) and primers GHR-For 5'-AGCCATTCATGGCAACTATAAATC-3', and GHR-Rev 5'-ARGGCAAGGCAGTTGCTTGAG -3' (modifications from those previously published by [8]). PCR reactions were done from 50 - 100 ng DNA using 1 unit of Supertherm Taq polymerase (Southern Cross Biotechnologies) in a 50 μl mixture consisting of 1× reaction buffer, 2.5 mM MgCl2 and 2 mM of each dNTP, 10 pM of each primer and ddH2O under the following cycling conditions: initial denaturation for 2 min at 94°C; 35 cycles of denaturation (30 sec) at 94°C, primer-annealing at 60 - 62°C (30 sec), primer-extension (45 sec) at 72°C; final extension of 5 min at 72°C. The resulting products were purified using isopropanol/ammonium acetate precipitation and were sequenced in both directions on an automated ABI 3100 sequencer (Applied Biosystems, Johannesburg, South Africa) using ABI PRISM Big DyeTM Terminator version 3.1 chemistry (Applied Biosystems). Nucleotide sequence alignments were constructed in ClustalX (v. 1.82 [33]) and subsequently translated to amino acids in MacClade (v. 3.0 [34]) to verify the functional reading frame. The GHR alignment of specimens amplified from both museum skulls and tissue samples comprised a matrix of up to 913 aligned nucleotides, of which 211 were parsimony informative [see Additional file 1].

Discrete morphological characters based on the matrix of Asher and Hofreiter [20] were collected using museum collections in Pretoria, Cape Town, King William's Town, Cambridge, London, Berlin, Stockholm, and New York. A total of 337 character states are distributed across 144 characters [see Additional file 3]. Of these characters, 45 are postcranial, 37 derive from the dentition and mandible, and 62 are from other parts of the cranium. These characters are graphically documented at http://www.morphobank.org[35]. Indels were recorded as in [20] and were entered as binary characters following the GHR alignment [see Additional file 1]. Gaps within the GHR alignment were treated as missing data.

Parsimony (MP) analyses were undertaken with PAUP 4.0b10 [36], using heuristic searches with at least 500 random addition replicates using TBR branch swapping, multiple states treated as polymorphic, and branches collapsed unless they have at least one unambiguous optimization ("COLLAPSE = MINBRLEN"). All morphological character state changes were accorded equal weight. MP bootstrap values were calculated based on 500 pseudoreplicates of a simple addition sequence. Analysis of the partitions shown in Table 1 was conducted using MP for each partition separately, using a heuristic search as above but with 100 random addition replicates.

Bayesian analyses were implemented using MrBayes 3.1 [37], using the HKY+G model as recommended by the AIC in MrModeltest 2.1 [38]. We used the default models for our morphological (i.e., Mk [39]) and indel (i.e., restriction site [37]) partitions, setting MrBayes to infer coding bias assuming that only variable characters can be observed for both ("CODING = VARIABLE"). Additional analyses assuming "CODING = ALL" for the morphological partition did not appreciably change the optimal topologies. Bayesian analyses were undertaken using four independent runs, each using a random starting tree and 1,000,000 generations with one cold and three heated chains, sampling trees every 100 generations. These reached stationarity by 10,000 generations (i.e., after the first 100 trees), at which point likelihood scores reached an asymptote and did not greatly change in value. Hence, Bayesian trees and posterior probabilities derive from a majority-rule consensus of the last 9901 trees for the first of the four independent runs, ignoring the first 100 as "burn-in". In each case, the four runs of 1,000,000 generations converged on a consistent topology. Likelihood analysis (including bootstraps) of the GHR data alone also used PAUP [36] and the HKY+G model as estimated by the AIC in MrModeltest [38], i.e., transition/transversion ratio = 2.2696, nucleotide frequencies A = 0.2805, C = 0.265, G = 0.2218, T = 0.2327, assumed proportion of invariable sites = none, distribution of rates at variable sites = gamma, shape parameter = 0.8383.

In order to investigate the part(s) of the chrysochlorid tree most attracted to a long branch, we followed a method described by Sullivan and Swofford [21]. That is, we generated 100 artificial taxa, composed of 913 randomly picked nucleotides (consistent with the number of aligned nucleotides and base frequencies [A 28%, C 27%, G 22%, T 23%] observed in the outgroup taxon *Elephantulus *[similar to other potential outgroups], chosen arbitrarily among afrotherian taxa known to be close to chrysochlorids), followed by randomly generated morphological (145) and indel (8) characters, all of which were binary. Each of these randomly generated "outgroups" was successively used as the root for an MP analysis sampling our 18 chrysochlorids, preserving unchanged their GHR, morphology, and indel characters. MP searches were heuristic, as described above, but with 25 random addition replicates. The position of the root was noted in each of the 100 simulations. For those analyses that did not yield a resolved root in the strict consensus, a 50% majority rule consensus was used (13 cases). Seven out of 100 cases did not yield a resolved root using majority rule and were ignored.

## Authors' contributions

RJA contributed to data acquisition and wrote the paper. All authors contributed to research design; SM, GB, and MH contributed to data acquisition; GB, PB and NB obtained funding for collection of South African samples for molecular data acquisition. GB contributed to morphological data coding, analysis, and interpretation, SM and PB commented on molecular data analysis and interpretation, PB and NB contributed to earlier drafts of the manuscript with respect to data interpretation. All authors have read and approved the final manuscript.

## Supplementary Material

Additional file 1 **Combined data nexus file**. GHR alignment, indel, and morphological character data, plus 8 optimal MP trees and MrBayes command block, in nexus format, entitled "gmole-comb-feb10.nex". Click here for file

Additional file 2 **Table of Blast results**. BLAST results for GHR sequences obtained from museum skulls. All fragments recovered maximum similarity to GHR sequences of *Chrysospalax trevelyan*i (AF392877 [30]). Click here for file

Additional file 3 **Morphological data nexus file**. Morphological characters with character and state names in nexus format entitled "gmole-morph-feb10.nex". Click here for file
