# Supplementary material for: A phylogenetic estimate for golden moles (Mammalia, Afrotheria, Chrysochloridae)
Source: BMC Evol Biol. 2010 Mar 9;10:69. doi: 10.1186/1471-2148-10-69 (PMC2850353; doi:10.1186/1471-2148-10-69)
Supplement: Additional file 2 — Table of Blast results. BLAST results for GHR sequences obtained from museum skulls. All fragments recovered maximum similarity to GHR sequences of Chrysospalax trevelyani (AF392877 [30]). [file 1471-2148-10-69-S2.DOC]

**Additional file 2.**

BLAST results for GHR sequences obtained from museum skulls. All fragments recovered maximum similarity to GHR sequences of *Chrysospalax trevelyan*i (AF392877 [Error: Reference source not found]).

| **taxon** | **source** | **fragment** | **length (bp)** | **% similarity to AF392877** |
| --- | --- | --- | --- | --- |
| *C. stuhlmanni* | ZMB 29456 | 1 | 114 | 99 |
|  |  | 2 | 104 | 98 |
|  |  | 3 | 120 | 99 |
|  |  | 4 | 135 | 95 |
|  |  | 5 | 124 | 96 |
|  |  | 6 | 118 | 100 |
|  |  | 7 | 120 | 98 |
|  |  | 8 | 111 | 95 |
|  |  | 9 | 137 | 96 |
|  |  | 10 | 135 | 98 |
|  |  | 11 | 126 | 91 |
|  |  | 12 | 160 | 92 |
| *C. wintoni* | TM 8235 | 1 | 114 | 100 |
|  |  | 2 | 104 | 100 |
|  |  | 3 | 120 | 100 |
|  |  | 4 | 135 | 97 |
|  |  | 5 | 124 | 97 |
|  |  | 6 | 118 | 99 |
|  |  | 7 | 120 | 98 |
|  |  | 8 | 111 | 98 |
|  |  | 9 | 137 | 98 |
|  |  | 10 | 135 | 97 |
|  |  | 11 | 90 | 93 |
|  |  | 12 | 128 | 95 |
|  |  | 13 | 117 | 93 |
|  |  | 14 | 160 | 95 |
| *E. granti* | TM 8248 | 1 | 114 | 100 |
|  |  | 2 | 104 | 100 |
|  |  | 3 | 135 | 97 |
|  |  | 4 | 124 | 97 |
|  |  | 5 | 118 | 98 |
|  |  | 6 | 120 | 98 |
|  |  | 7 | 111 | 98 |
|  |  | 8 | 135 | 97 |
|  |  | 9 | 78 | 95 |
|  |  | 10 | 117 | 94 |
|  |  | 11 | 113 | 97 |
| *A. hottentotus* | ZMB 3919 | 1 | 114 | 100 |
|  |  | 2 | 104 | 100 |
|  |  | 3 | 135 | 97 |
|  |  | 4 | 124 | 96 |
|  |  | 5 | 118 | 99 |
|  |  | 6 | 120 | 98 |
|  |  | 7 | 111 | 100 |
|  |  | 8 | 137 | 98 |
|  |  | 9 | 135 | 98 |
|  |  | 10 | 90 | 97 |
| **taxon** | **source** | **fragment** | **length (bp)** | **% similarity to AF392877** |
|  |  | 11 | 126 | 96 |
|  |  | 12 | 117 | 95 |
|  |  | 13 | 160 | 96 |
|  |  | 14 | 113 | 97 |
| *C.obtusirostris* | ZMB 12945 | 1 | 120 | 100 |
|  |  | 2 | 124 | 97 |
|  |  | 3 | 116 | 100 |
|  |  | 4 | 118 | 100 |
|  |  | 5 | 120 | 98 |
|  |  | 6 | 111 | 99 |
|  |  | 7 | 137 | 99 |
|  |  | 8 | 135 | 99 |
|  |  | 9 | 90 | 97 |
|  |  | 10 | 128 | 98 |
|  |  | 11 | 126 | 98 |
|  |  | 12 | 117 | 97 |
|  |  | 13 | 114 | 97 |
|  |  | 14 | 125 | 97 |
